# Data-driven parcellation and graph theory analyses to study adolescent mood and anxiety symptoms

**DOI:** 10.1038/s41398-021-01321-x

**Published:** 2021-05-03

**Authors:** Benjamin A. Ely, Qi Liu, Samuel J. DeWitt, Lushna M. Mehra, Carmen M. Alonso, Vilma Gabbay

**Affiliations:** 1 Department of Psychiatry and Behavioral Sciences, Albert Einstein College of Medicine, Bronx, NY USA; 2 Department of Psychiatry, Icahn School of Medicine at Mount Sinai, New York, NY USA; 3 Department of Psychology, Florida State University, Tallahassee, FL USA; 4 Nathan S. Kline Institute for Psychiatric Research, Orangeburg, NY USA

**Keywords:** Neuroscience, Depression, Diagnostic markers

## Abstract

Adolescence is a period of rapid brain development when psychiatric symptoms often first emerge. Studying adolescents may therefore facilitate the identification of neural alterations early in the course of psychiatric conditions. Here, we sought to utilize new, high-quality brain parcellations and data-driven graph theory approaches to characterize associations between resting-state networks and the severity of depression, anxiety, and anhedonia symptoms—salient features across psychiatric conditions. As reward circuitry matures considerably during adolescence, we examined both *Whole Brain* and three task-derived reward networks. Subjects were 87 psychotropic-medication-free adolescents (age = 12–20) with diverse psychiatric conditions (*n* = 68) and healthy controls (*n* = 19). All completed diagnostic interviews, dimensional clinical assessments, and 3T resting-state fMRI (10 min/2.3 mm/TR = 1 s). Following high-quality Human Connectome Project-style preprocessing, multimodal surface matching (MSMAll) alignment, and parcellation via the Cole-Anticevic Brain-wide Network Partition, weighted graph theoretical metrics (Strength Centrality = C_Str_; Eigenvector Centrality = C_Eig_; Local Efficiency = E_Loc_) were estimated within each network. Associations with symptom severity and clinical status were assessed non-parametrically (two-tailed *p*_*FWE*_ < 0.05). Across subjects, depression scores correlated with ventral striatum C_Str_ within the *Reward Attainment* network, while anticipatory anhedonia correlated with C_Str_ and E_Loc_ in the subgenual anterior cingulate, dorsal anterior cingulate, orbitofrontal cortex, caudate, and ventral striatum across multiple networks. Group differences and associations with anxiety were not detected. Using detailed functional and clinical measures, we found that adolescent depression and anhedonia involve increased influence and communication efficiency in prefrontal and limbic reward areas. Resting-state network properties thus reflect positive valence system anomalies related to discrete reward sub-systems and processing phases early in the course of illness.

## Introduction

Adolescence represents a critical period of development during which many prodromal psychiatric symptoms and conditions first emerge, including depression, anxiety, and substance abuse^1^. This increased incidence has been attributed to rapid maturational changes in the brain during adolescence, which involve synaptic pruning, myelination, neurotransmission, and the formation of mature intrinsic functional circuits found in adults^2–4^. Specifically, adolescence is a period of time when reward-seeking behaviors are dominant, with reward circuitry undergoing major changes in corticolimbic and frontal regions^5^. As such, deviations from normal developmental processes in the reward system are hypothesized to underlie the emergence of psychiatric conditions in adolescence, and studying adolescents may therefore facilitate the identification of modifiable factors early in the course of psychiatric conditions. A persistent challenge in delineating the neural underpinnings of mental illness has been that psychiatric diagnoses are based on clusters of symptoms with distinct etiology and high comorbidity^6^. In response, our group and others have increasingly focused on specific symptoms^7–9^, which represent narrowly defined clinical features with potentially distinct etiologies, rather than broad categorical diagnoses. In this study, we sought to utilize such a dimensional approach to examine the neural correlates of anxiety, anhedonia, and overall depression severity in adolescents with diverse psychiatric conditions.

This study further employed a data-driven analysis approach based on graph theory, which models complex systems like the brain as collections of nodes (e.g., cortical areas) linked by edges (e.g., functional connectivity)^10^. Graph theory provides a concise way of defining and representing resting-state networks, and can reveal subtle network features that compliment and transcend the information provided by traditional connectivity analyses. Important graph theoretical metrics include nodal measures of centrality (i.e., influence over other nodes) and efficiency (i.e., ease of communication with other nodes)^10^. Our work also builds on the recent advances in neuroimaging methodology spearheaded by the Human Connectome Project (HCP)^11^. In 2016, the HCP released a landmark parcellation identifying 360 distinct cortical areas based on multimodal measures of cortical thickness, myelination, resting-state functional connectivity, and task activation patterns in an extensively sampled cohort of healthy young adults^12^. Recently, the Cole-Anticevic Brain-wide Network Partition (CAB-NP) has extended this parcellation scheme to include the subcortex, identifying 358 further regions on the basis of resting-state network assignments and providing a detailed map of discrete functional areas across the entire brain^13^. In addition to revealing fundamental aspects of neural organization, high-quality parcellations provide an invaluable framework for further data-driven research. The principled data reduction enabled by these parcellations is especially crucial in graph theory, which has been limited in past studies by the use of overly simplistic network models and functionally inaccurate node boundaries^14,15^. To date, only a handful of studies have employed these new, rigorously defined parcellation maps to examine psychiatric conditions.

Building upon the developments described above, our aim was to use high-quality parcellations and detailed network models to examine the neural correlates of depression, anhedonia, and anxiety, assessed quantitatively in psychotropic-medication-free adolescents with diverse psychiatric symptoms. As clinical symptomatology is salient across disorders and lies on a continuum within each disorder, our study was designed to capture the full range of symptom severity by recruiting a large, transdiagnostic sample that included adolescents with comorbid and subthreshold diagnoses as well as healthy controls. Using graph theory, we examined relationships between clinical symptomatology and resting-state network properties of centrality and efficiency within the functionally accurate CAB-NP network^13^. As reward circuitry plays a central role in the emergence of psychiatric conditions during adolescence, we also repeated analyses within three functionally defined reward networks derived from the Reward Flanker Task (RFT)^16^. We hypothesized that resting-state network properties in regions related to reward and aversion processing would be associated with anhedonia and anxiety severity, respectively, and that both sets of regions would be associated with overall depression severity and clinical status.

## Subjects and methods

### Recruitment

Adolescents, ages 12–20, were recruited from the greater New York City area. The study was approved by the Institutional Review Board at Icahn School of Medicine at Mount Sinai. Prior to the study, procedures were explained to adolescents and legal guardians. Participants age 18+ provided written consent; those under 18 provided written assent and a guardian provided written consent.

### Inclusion and exclusion criteria

#### All participants

Adolescents were excluded if they had any significant medical or neurological condition, estimated IQ < 80, claustrophobia, any MRI contraindication, or a positive urine toxicology or pregnancy test.

#### Psychiatric group

Clinical participants were psychotropic-medication-free for 30+ days, or 90+ days for long half-life medications (e.g., fluoxetine). Exclusionary diagnoses were pervasive developmental disorders, current psychosis, or a substance use disorder in the past year. All other psychiatric conditions were allowed, regardless of whether full diagnostic criteria were met.

#### Healthy control group

Control participants did not meet the criteria for any current or past psychiatric diagnoses and were psychotropic-medication-naïve.

### Clinical measures

#### Diagnostic procedures

Clinical and sub-clinical DSM-IV-TR diagnoses were obtained using the Schedule for Affective Disorders and Schizophrenia—Present and Lifetime Version (K-SADS-PL)^17^. Interviews were administered to all adolescent participants without guardians present, as well as to guardians without adolescents present for participants under age 18. Evaluations were discussed between the interviewing clinician and Principal Investigator (VG), a board-certified child and adolescent psychiatrist, in order to enhance reliability.

#### Depression

Overall depression severity was assessed using the Beck Depression Inventory-II (BDI), a 21-item scale that assesses symptoms and features of depression over the previous 2 weeks and has high internal consistency in both clinical and non-clinical adolescent populations^18^.

#### Anhedonia

Anhedonia severity was assessed by the state-based Temporal Experience of Pleasure Scale (TEPS). This 18-item self-report separately quantifies anticipatory (TEPS-A) and consummatory (TEPS-C), as well as total (TEPS-T), anhedonia symptoms over the past week^19^. Since the TEPS is reverse-scored (higher scores→lower anhedonia), analyses were performed using negative TEPS values (higher scores→higher anhedonia) for consistency with other scales.

#### Anxiety

Anxiety severity was examined using the Multidimensional Anxiety Scale for Children (MASC), a 39-item scale validated in both clinical and non-clinical populations^20^.

### Imaging data acquisition

Data were acquired on a 3T Skyra MR system (Siemens, Germany) with 16/4-channel head/neck coil using protocols similar to the HCP Lifespan study^21^. Sequences included: T1-weighted MPRAGE (TR = 2400 ms; TE = 2.06 ms; TI = 1000 ms; flip angle = 8°; 224 sagittal frames, no gap; matrix = 256 × 256; FOV = 230 × 230mm^2^; 0.9 mm isotropic), T2-weighted SPACE (TR = 3200 ms; TE = 565 ms; flip angle=120°; 224 sagittal frames, no gap; matrix = 256 × 256; FOV = 230 × 230mm^2^; 0.9 mm isotropic), and resting-state gradient-recalled EPI (10 min; TR = 1000 ms; effective TE = 31.4 ms; flip angle = 60°; 600 frames of 60 slices parallel to AC-PC, no gap; 5× multiband acceleration; anterior-to-posterior phase encoding; matrix = 98 × 98; FOV = 228 × 228 mm^2^; 2.3 mm isotropic). Matched single-band EPI and spin-echo fieldmaps were collected for registration and distortion correction purposes. Subjects were presented with a fixation cross and instructed to rest with their eyes open. Four RFT fMRI runs (6 min 14 s each) were also acquired later in the session using similar sequences.

### Imaging data processing

Data were visually inspected before preprocessing with HCP Pipelines v3.4^22^. For anatomical data, preprocessing included gradient nonlinearity correction, b_0_ distortion correction, AC-PC alignment, coregistration, brain extraction, bias-field correction, nonlinear transformation to MNI space, FreeSurfer segmentation, and cortical ribbon extraction. Functional data were corrected for gradient nonlinearity and EPI readout distortion, realigned, transformed to MNI space, intensity normalized, and initially mapped to the cortical ribbon using default FreeSurfer alignment. Subject-level dense timeseries were generated in 32k-CIFTI grayordinate space, which combines functional data from left and right 2D cortical surfaces with major subcortical structures in 3D MNI space to accurately represent gross brain anatomy^22^.

Next, structured fMRI noise components were automatically identified and removed using the multi-run implementation of spatial ICA-FIX developed by the HCP^23,24^. This version achieves excellent denoising performance, comparable to the original single-run ICA-FIX, but can accommodate shorter fMRI scans by using concatenated data from multiple resting-state and/or task runs^25^. Combined subject-level fMRI data consisted of the single resting-state run (600 frames) and 2–4 RFT runs (374 frames each). Runs with excessive motion, defined as ≥5% of frames with mean framewise displacement ≥1 mm, were excluded. Concatenated subject-level fMRI data were mildly high-pass filtered (default 2000s cutoff). Whole-brain MNI timeseries were then decomposed into independent components (ICs) via the FSL MELODIC tool^26^. Each IC was automatically classified as “signal”, “noise”, or “unknown” via the FIX classifier algorithm (default HCP_hp2000.Rdata training set), which we have previously benchmarked as achieving >97% accuracy in locally acquired fMRI datasets. All “signal” and “unknown” ICs were jointly reviewed by two experienced neuroimagers (B.A.E. and Q.L.) and manually reclassified as necessary. “Noise” ICs were then removed from both the MNI- and grayordinate space fMRI data using “soft” regression (i.e., only unique variance removed).

Following ICA-FIX, cortical surface data in grayordinate space were robustly aligned across subjects based on a combination of functional and anatomical features using the multimodal surface matching (MSMAll) method developed and advocated by the HCP^27,28^. MSMAll was performed using all low-movement resting-state and RFT fMRI data included in the multi-run ICA-FIX denoising step.

In addition to ICA-FIX denoising, we performed global signal regression (GSR) to minimize the effects of respiratory-related intensity fluctuations and other global sources of residual noise^29,30^. Mean whole-brain timeseries were extracted from FIX-denoised resting-state fMRI in MNI space using Conn Toolbox v17f^31^ and removed using “hard” regression (i.e., all variance removed) from the FIX-denoised, MSMAll-aligned fMRI data in grayordinate space. In light of longstanding debates over the benefits vs. drawbacks of GSR^25,32^, all analyses were also performed without including this step (see Supplementary Results). No spatial smoothing or bandpass filtering was applied.

**Fig. 1:**
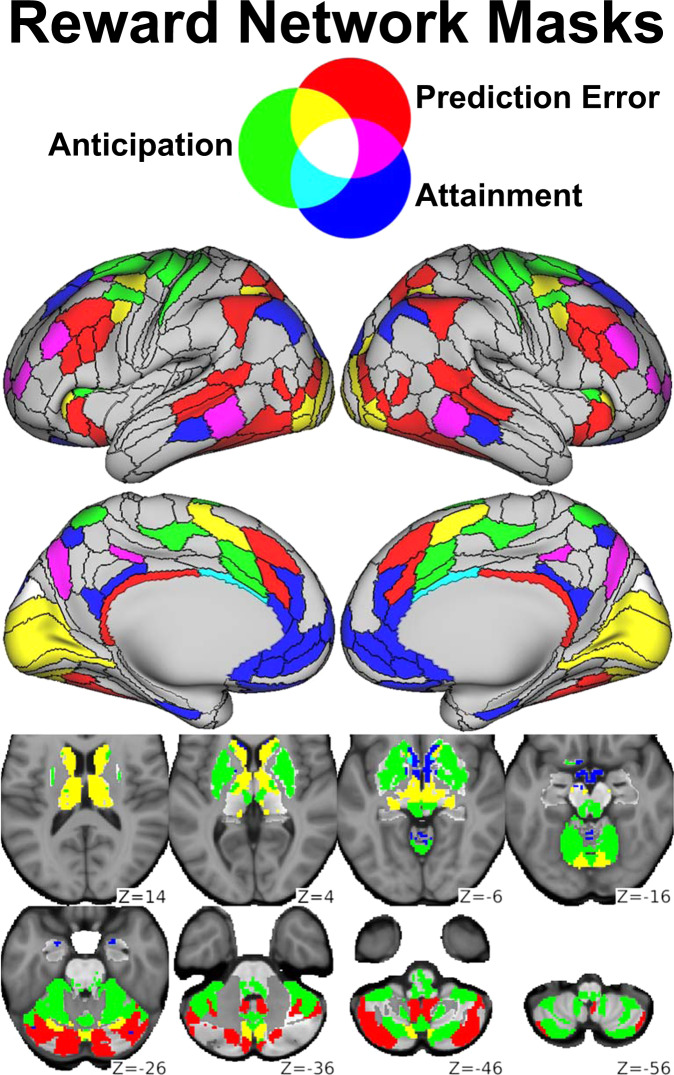
Reward network masks. Nodes from the *Whole Brain* network corresponding to *Reward Anticipation* (green), *Reward Attainment* (blue), and *Reward Prediction Error* (red) networks derived from the Reward Flanker Task (RFT). Nodes included in multiple networks are indicated by additive color mixing, as shown at the top.

Finally, denoised resting-state fMRI data in grayordinate space were parcellated (i.e., divided into nodes and spatially averaged within each) using CAB-NP v1.0.5, which extends the HCP cortical parcellation^12^ to include functionally similar subcortical parcels^13^. As in previous work^33^, we slightly modified the cortical component of this parcellation by subdividing the somatomotor strip along somatotopic boundaries, yielding the final *Whole Brain* network (750 nodes). In addition, we identified three reward-related networks (Fig. 1) based on a separate analysis of RFT fMRI data collected in the same sample (manuscript in preparation), which builds on our previous RFT studies^16,34^. Briefly, these networks comprised the 10% of nodes most activated by *Reward Anticipation* (114 nodes), *Reward Attainment* (103 nodes), and *Reward Prediction Error* (117 nodes) RFT contrasts, as well as any corresponding contralateral nodes; see Supplementary Methods for additional details.

### Graph theoretical metrics

Subject-level association matrices were generated by cross-correlating node timeseries within the *Whole Brain* (750 × 750), *Reward Anticipation* (114 × 114), *Reward Attainment* (103 × 103), and *Reward Prediction Error* (117 × 117) networks in MATLAB v2017a and retaining all positive *r* values. Within each network, graph theoretical metrics were then estimated using weighted, undirected measures from Brain Connectivity Toolbox v2019–03–03^10^.

1. Strength Centrality (C_Str_): The sum of all edge weights (i.e., positive *r* values) at each node. C_Str_ is the weighted analog of the binary Degree Centrality metric.
2. Eigenvector Centrality (C_Eig_): The eigenvector with the largest eigenvalue for each node. This measurement is self-referential, such that nodes with high C_Eig_ are those most closely associated with other high-C_Eig_ nodes.
3. Local Efficiency (E_Loc_): The inverse shortest path length (i.e., minimum number of edges, adjusted for edge weights) between each node and its neighborhood.

### Graph theory analysis

Group differences (clinical vs. control) in graph theoretical metrics were assessed using two-sample, unequal-variance *t*-tests. Relationships between graph theoretical metrics and symptom scales (BDI, MASC, negative TEPS) were assessed using Pearson partial correlations in the full sample. All analyses controlled for participant age and sex. Statistical significance was determined using non-parametric permutation tests (10,000 iterations), as implemented in FSL PALM v111-alpha^35^. Non-parametric tests provide better familywise error (FWE) control than their parametric equivalents^36^ and are robust to skewed data distributions^37^, as was the case for symptom scales in our study (skewness: BDI = 1.32; MASC = 0.58; TEPS-A = −0.94; TEPS-C = −0.75; TEPS-T = −1.00). Results were considered significant at the two-tailed *p*_*FWE*_ < 0.05 level. Given our sample characteristics (see “Results” section), sensitivity analyses indicated ~80% power to detect effect sizes of |d| ≥0.74 for group differences and |r| ≥ 0.29 for symptom correlations, consistent with our findings.

## Results

### Clinical characteristics

The sample included 87 adolescents, of whom 68 had psychiatric symptoms (predominantly related to mood and anxiety) and 19 were healthy controls. Table 1 provides participant demographic and clinical characteristics. Relative to controls, adolescents with psychiatric symptoms had significantly higher BDI and MASC scores (*p*_*FWE*_ < 10^–3^). Groups did not differ significantly in age, sex, race, ethnicity, or TEPS scores (*p*_*FWE*_ > 0.1).

**Table 1:** Clinical and demographic information.

Measure	Control (*n* = 19)	Clinical (*n* = 68)	All (*n* = 87)
Age (M ± SD)	15.3 ± 2.5	15.1 ± 2.1	15.2 ± 2.2
Sex	F = 9, M = 10	F = 44, M = 24	F = 53, M = 34
Race^a^	Af = 8, As = 0, E = 7, O = 4	Af = 24, As = 2, E = 30, O = 12	Af = 32, As = 2, E = 37, O = 16
Ethnicity^b^	*H* = 5, *N* = 14	*H* = 33, *N* = 35	*H* = 38, *N* = 49
BDI (M ± SD)	1.8 ± 2.1 (*n* = 19)	13.6 ± 11.4 (*n* = 66)	11.0 ± 11.2 (*N* = 85)
MASC (M ± SD)	27.0 ± 11.7 (*n* = 17)	44.7 ± 17.3 (*n* = 66)	41.1 ± 17.8 (*N* = 83)
TEPS-A (M ± SD)	49.4 ± 6.8 (*n* = 18)	45.0 ± 8.9 (*n* = 52)	46.1 ± 8.6 (*N* = 70)
TEPS-C (M ± SD)	35.0 ± 8.7 (*n* = 18)	33.2 ± 7.5 (*n* = 52)	33.6 ± 7.8 (*N* = 70)
TEPS-T (M ± SD)	84.4 ± 13.7 (*n* = 18)	78.1 ± 14.5 (*n* = 52)	79.7 ± 14.5 (*N* = 70)
Mood symptoms^c^	0	49	49
Anxiety symptoms^c^	0	43	43
Behavioral symptoms^c^	0	28	28
Other symptoms^c^	0	7	7

^a^*Af* = African American, *As* = Asian American, *E* = European American, *O* = Other/Mixed Race.

^b^*H* = Hispanic, *N* = Non-Hispanic.

^c^Includes past and/or subthreshold symptoms.

### Group differences

No significant differences in graph theoretical metrics were observed between adolescents with psychiatric symptoms and healthy controls for any network in the main analysis. In the repeated analysis without GSR (Supplementary Results), *Reward Anticipation* network C_Str_, C_Eig_, and E_Loc_ in frontal language area 55b were elevated for clinical subjects relative to controls (Table S1); no other significant group differences were observed.

### Depression severity

**Fig. 2:**
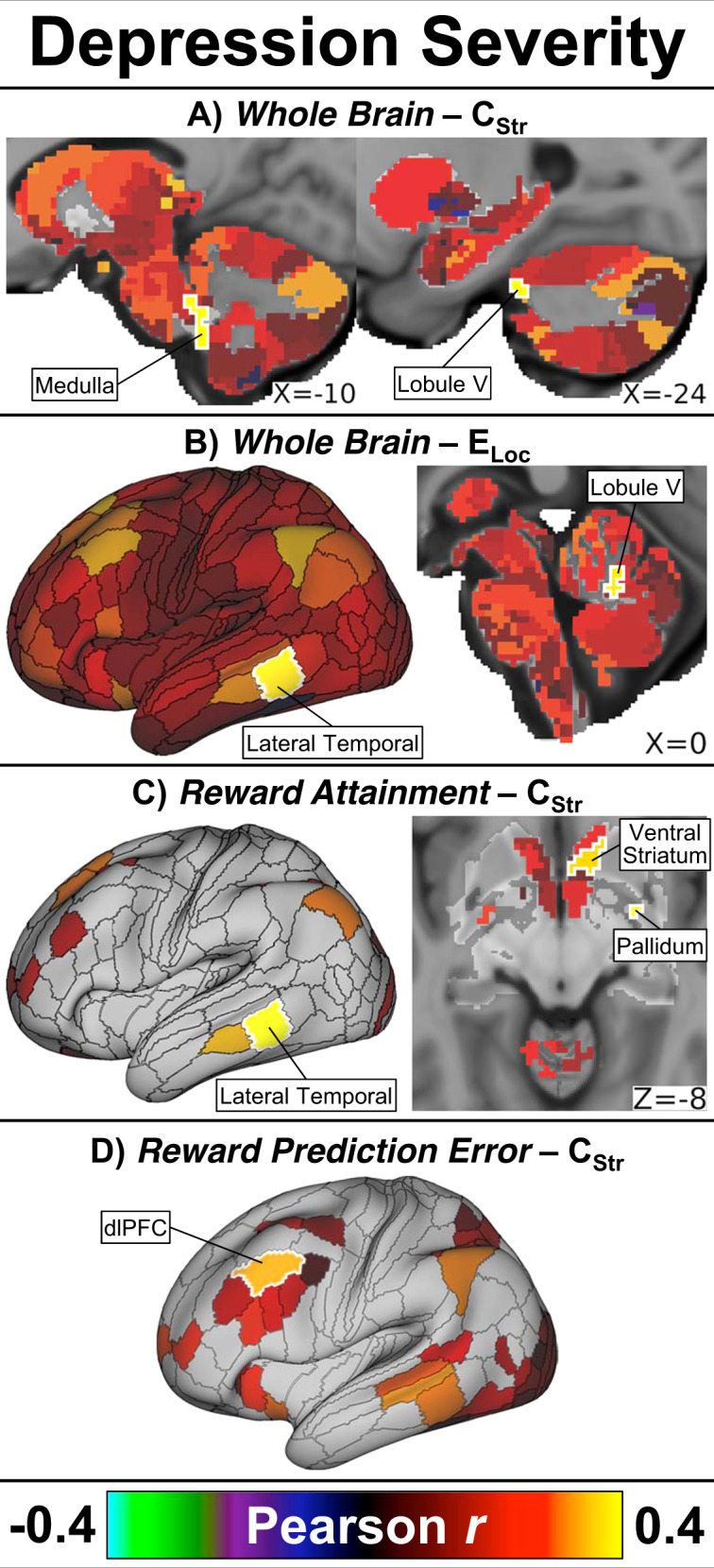
Depression severity correlation results. Across all adolescents, overall depression severity positively correlated with: **a** *Whole Brain* C_Str_ in the left medullary brainstem and left cerebellum; **b** *Whole Brain* E_Loc_ in the left lateral temporal cortex and left cerebellum; **c** *Reward Attainment* network C_Str_ in the left lateral temporal cortex, right ventral striatum, and right pallidum; and **d** *Reward Prediction Error* network C_Str_ in the left dorsolateral prefrontal cortex (dlPFC). Maps show effect size (Pearson’s *r*) adjusted for age and sex. Significant nodes (two-tailed *p*_*FWE*_ < 0.05) are indicated by white outlines and labels; non-significant nodes are displayed at 50% saturation.

Depression severity (BDI) was positively correlated with: *Whole Brain* C_Str_ in the left medulla and cerebellum; *Whole Brain* E_Loc_ in the left lateral temporal lobe and cerebellum; *Reward Attainment* network C_Str_ in two right ventral striatum nodes and the left lateral temporal lobe; *Reward Attainment* network C_Str_ and E_Loc_ in the right inferior pallidum; *Reward Attainment* network C_Str_, C_Eig_, and E_Loc_ in a small left medial hippocampus node; and *Reward Prediction Error* network C_Str_ in the left dorsolateral prefrontal cortex (dlPFC). No associations with depression were detected within the *Reward Anticipation* network. Select findings are displayed in Fig. 2, with full results detailed in Table 2. In the repeated analysis without GSR, only depression correlations with *Reward Attainment* network C_Str_ and E_Loc_ in the right ventral striatum and inferior pallidum remained significant (Table S2).

**Table 2:** Graph theory symptom correlation results.

Network	Metric	Location	CAB-NP Label (HCP Cortical Label)^a^	Pearson *r*^b^	*p*_*FWE*_
Depression severity					
*Whole Brain*	C_Str_	Left medullary brainstem	Frontoparietal-6	0.400	0.041
		Left cerebellum lobule V	Frontoparietal-17	0.416	0.027
		Left cerebellum lobule V	Frontoparietal-21	0.395	0.048
	E_Loc_	Left lateral temporal	Frontoparietal_44 (TE1p)	0.375	0.049
		Left cerebellum lobule V	Frontoparietal-17	0.381	0.042
		Left cerebellum lobule V	Frontoparietal-18	0.379	0.044
*Reward Attainment*	C_Str_	Left lateral temporal	Frontoparietal_44 (TE1p)	0.434	0.0042
		Left medial hippocampus	Default-24	0.426	0.0049
		Right inferior pallidum	Orbito-Affective-17	0.419	0.0060
		Right ventral striatum	Cingulo-Opercular-3	0.372	0.031
		Right ventral striatum	Orbito-Affective-2	0.362	0.041
	C_Eig_	Left medial hippocampus	Default-24	0.375	0.033
	E_Loc_	Right inferior pallidum	Orbito-Affective-17	0.416	0.0061
		Left medial hippocampus	Default-24	0.441	0.0026
*Reward Prediction Error*	C_Str_	Left dlPFC	Frontoparietal_32 (8C)	0.326	0.048
Anticipatory anhedonia severity					
*Whole Brain*	E_Loc_	Left lateral parietal	Default_68 (PGi)	0.418	0.043
		Left posterior lateral temporal	Visual2_44 (PH)	0.428	0.032
		Left superior parietal	Visual2_43 (VIP)	0.414	0.048
		Left superior parietal	Visual2_35 (IPS1)	0.461	0.012
		Left lateral temporal	Ventral_Multimodal_3 (TF)	0.423	0.037
		Left sgACC	Default_74 (s32)	0.417	0.045
		Right lateral occipital	Visual2_12 (LO2)	0.417	0.045
		Right lateral temporal	Default_36 (STSva)	0.421	0.040
		Right temporal pole	Default_27 (TGd)	0.434	0.027
		Right parahippocampus	Default_32 (PHA2)	0.453	0.015
		Right parahippocampus	Dorsal_Attention_6 (PHA3)	0.417	0.045
		Left pulvinar thalamus	Auditory-24	0.441	0.022
		Left medial hippocampus	Default-24	0.434	0.027
		Inferior medial brainstem	Visual-6	0.465	0.010
*Reward Anticipation*	E_Loc_	Left caudate	Frontoparietal-10	0.365	0.044
		Right caudate	Frontoparietal-11	0.381	0.028
*Reward Attainment*	C_Str_	Left lateral temporal	Frontoparietal_44 (TE1p)	0.405	0.033
	E_Loc_	Left sgACC	Default_74 (s32)	0.421	0.019
		Right dACC	Frontoparietal_4 (d32)	0.430	0.015
		Right OFC	Frontoparietal_15 (13l)	0.392	0.044
		Left medial hippocampus	Default-24	0.411	0.025
*Reward Prediction Error*	C_Str_	Left caudate	Frontoparietal-10	0.381	0.035
		Right caudate	Frontoparietal-11	0.408	0.017
		Left superior parietal	Dorsal_Attention_13 (MIP)	0.377	0.038
	E_Loc_	Right ventral striatum	Cingulo-Opercular-11	0.377	0.044
		Left caudate	Frontoparietal-10	0.418	0.011
		Right caudate	Frontoparietal-11	0.435	0.0066
		Right thalamus	Visual-63	0.387	0.031
		Right dACC	Cingulo-Opercular_27 (a32pr)	0.383	0.037
		Right dlPFC	Frontoparietal_12 (a9–46v)	0.424	0.010
Total anhedonia severity					
*Whole Brain*	E_Loc_	Left lateral temporal	Ventral_Multimodal_3 (TF)	0.425	0.036
		Right medial temporal	Default_32 (PHA2)	0.423	0.038
		Right hippocampus	Visual2–26	0.432	0.029
		Right hippocampus	Somatomotor-19	0.424	0.036
		Left hippocampus	Somatomotor-16	0.436	0.026
		Left medial hippocampus	Default-24	0.427	0.034
		Medial inferior brainstem	Visual-6	0.432	0.031
*Reward Prediction Error*	E_Loc_	Right caudate	Frontoparietal-11	0.375	0.049
		Left cerebellum crus II	Posterior Multimodal-13	0.384	0.036

^a^Labels per Cole-Anticevic Brain-wide Network Partition v1.0.5 (equivalent labels per HCP S1200 Release cortical parcellation).

^b^Adjusted for age and sex. Anhedonia correlations reported with negative TEPS for consistency with other scales (see “Methods” section).

### Anhedonia severity

**Fig. 3:**
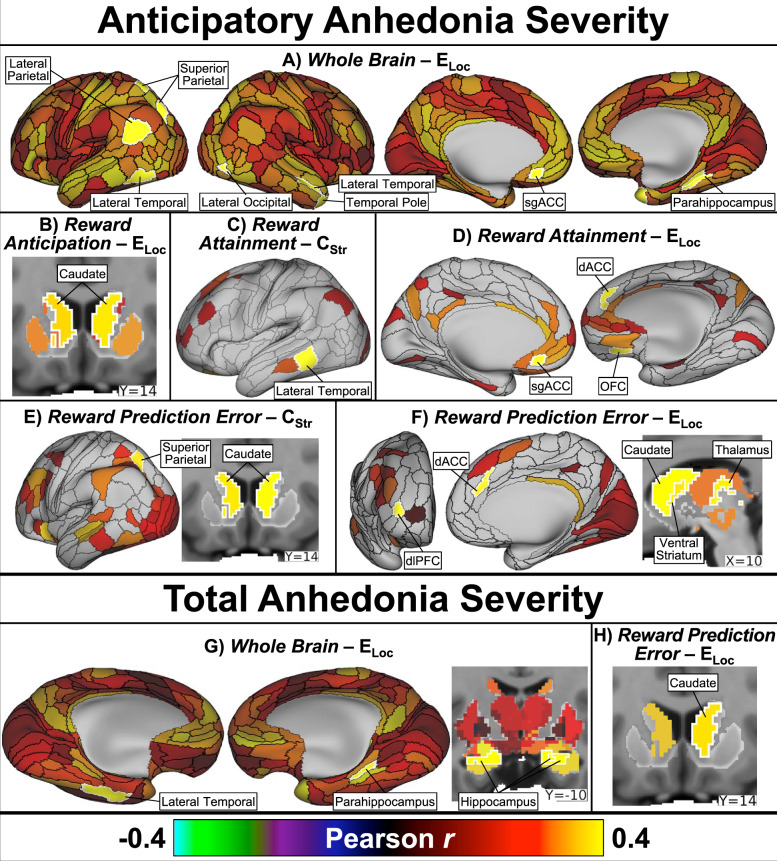
Anhedonia severity correlation results. Across all adolescents, anticipatory anhedonia positively correlated with: **a** *Whole Brain* E_Loc_ in the left lateral and superior parietal cortices, bilateral lateral temporal cortex, right temporal pole, right lateral occipital cortex, left subgenual anterior cingulate cortex (sgACC), and right parahippocampus; **b** *Reward Anticipation* network E_Loc_ in the bilateral caudate; **c** *Reward Attainment* network C_Str_ in the left lateral temporal cortex; **d** *Reward Attainment* network E_Loc_ in the left sgACC, right orbitofrontal cortex (OFC), and right dorsal anterior cingulate cortex (dACC); **e** *Reward Prediction Error* network C_Str_ in the left superior parietal cortex and bilateral caudate; and **f** *Reward Prediction Error* network E_Loc_ in the right dorsolateral prefrontal cortex (dlPFC), right dACC, bilateral caudate, right ventral striatum, and right thalamus. Total anhedonia positively correlated with: **g** *Whole Brain* E_Loc_ in the left lateral temporal cortex, right parahippocampus, and bilateral hippocampus; and **h** *Reward Prediction Error* network E_Loc_ in the right caudate. Maps show effect size (Pearson’s *r*) adjusted for age and sex. Significant (two-tailed *p*_*FWE*_ < 0.05) nodes are indicated by white outlines and labels; non-significant nodes are displayed at 50% saturation.

Anticipatory anhedonia (negative TEPS-A) was positively correlated with: *Whole Brain* E_Loc_ in the left subgenual anterior cingulate (sgACC), right parahippocampus, right temporal pole, bilateral lateral temporal lobe, left lateral and superior parietal cortices, and left pulvinar thalamus; *Reward Anticipation* network E_Loc_ in the bilateral caudate; *Reward Attainment* network C_Str_ in the left lateral temporal; *Reward Attainment* network E_Loc_ in the left sgACC, right orbitofrontal cortex (OFC), and right dorsal anterior cingulate (dACC); *Reward Prediction Error* network E_Loc_ in the right dACC and left dlPFC; and *Reward Prediction Error* network C_Str_ and E_Loc_ in the bilateral caudate. Total anhedonia (negative TEPS-T) was positively correlated with: *Whole Brain* E_Loc_ in the bilateral hippocampus, right parahippocampus, and left inferior lateral temporal lobe; and *Reward Prediction Error* network E_Loc_ in the right caudate. No associations with consummatory anhedonia (negative TEPS-C) were detected in the main analysis. Select findings are displayed in Fig. 3, with full results detailed in Table 2. The repeated analysis without GSR detected extensive (~200) associations with anhedonia across all three subscales; findings included the majority of nodes identified in the main analysis as well as numerous vision-related parietal and occipital areas (Table S2).

### Anxiety severity

No associations were detected between anxiety levels (MASC) and graph theoretical metrics for any network in either the main analysis or the supplementary analysis without GSR.

## Discussion

The present study capitalized on recent advances in neuroimaging methodology to examine resting-state network properties in the context of adolescent mental illness. Our approach included high-quality multiband fMRI sequences to achieve excellent spatial (2.3 mm isotropic) and temporal (1 s) resolution, HCP-style preprocessing including highly accurate MSMAll surface alignment, and a large sample of psychotropic-medication-free adolescents with diverse clinical symptomatology. A key element of our study was the CAB-NP parcellation, which enabled us to model networks using functionally discrete nodes across the entire cortex and subcortex. To further preserve neurobiological detail, we derived graph theoretical metrics of centrality (C_Str_, C_Eig_) and efficiency (E_Loc_) using weighted association matrices, rather than the simpler binary approach where association matrices are arbitrarily thresholded and all surviving correlations are treated as equivalent. In addition to *Whole Brain* analyses, we also examined graph theoretical metrics within specific *Reward Anticipation*, *Reward Attainment*, and *Reward Prediction Error* networks, which we defined empirically using task fMRI data collected in the same subjects. Importantly, these analyses within smaller RFT networks did not simply reduce multiple comparison penalties, as in small-volume correction^39^, but directly altered the calculation of graph theoretical metrics by restricting the underlying association matrix to nodes involved in the corresponding reward process.

As hypothesized, both anhedonia and overall depression severity correlation analyses implicated key reward-related areas, supporting the notion that alterations in reward circuitry during adolescent brain development play an important role in the emergence of psychiatric disorders. Across all adolescents, higher depression severity was associated with increased C_Str_ in the ventral striatum and pallidum within the *Reward Attainment* network, while higher anhedonia severity was associated with the increased C_Str_ or E_Loc_ in the sgACC, dACC, OFC, ventral striatum, and caudate across multiple networks. Taken together, these findings suggest that increased communication with reward areas during rest may be related to the initial development of positive valence system (PVS) deficits. Notably, analyses within the RFT-derived *Reward Anticipation*, *Reward Attainment*, and *Reward Prediction Error* networks revealed many further correlations between symptom severity and graph theoretical metrics than *Whole Brain* analyses alone. Moreover, our anhedonia findings were predominantly driven by anticipatory anhedonia, which involves undervaluation of expected rewards and is associated with motivational deficits, rather than consummatory anhedonia, which reflects the diminished experience of pleasure once rewards are obtained. As such, our results highlight the importance of studying specific reward sub-systems and considering discrete phases of reward processing, even at rest.

The benefits of this approach are evident in our depression severity correlation findings: while *Whole Brain* analyses implicated a few cerebellar and lateral temporal nodes, the same analyses within the more specific *Reward Attainment* network revealed further associations with C_Str_ in the right nucleus accumbens (NAc). The NAc and surrounding ventral striatum play a highly conserved role in primary reward processing, receiving dopaminergic inputs from the ventral tegmental area in response to appetitive stimuli via the mesolimbic reward pathway^40,41^. Similar to our result, a previous resting-state fMRI study in children aged 6–12 found that, within a network consisting of 12 reward-related nodes, only left ventral striatum C_Str_ significantly predicted the emergence of depression at 3-year follow-up^42^. Analyses within the *Reward Prediction Error* network, meanwhile, revealed a positive correlation between depression severity and C_Str_ in the left dlPFC, a region linked to therapeutic outcomes in depression. An early PET study in adults with severe depression found that reduced metabolic activity in the left dlPFC was associated with non-response to fluoxetine treatment^43^. Subsequently, multiple clinical trials have established transcranial magnetic stimulation of the left dlPFC as an effective non-pharmacological treatment for depression^44–46^. Our findings indicate that adolescent depression entails altered resting-state communication with these prefrontal and subcortical reward areas previously linked to depression chronicity and treatment response.

Beyond correlations with overall depression severity, our analyses revealed numerous associations between graph theoretical metrics and anhedonia, a core symptom of depression. In both the *Whole Brain* and *Reward Attainment* network analyses, anticipatory anhedonia positively correlated with E_Loc_ in the left sgACC, a prefrontal area important for sustaining arousal and positive affect in anticipation of expected rewards^47^. Increased sgACC activity is frequently reported in neuroimaging studies of depressed adults^48^ and adolescents^49^, while sgACC activity decreases following many types of depression treatment, including traditional antidepressants, ketamine, and deep brain stimulation^50–52^. In an earlier resting-state functional connectivity study of the striatum in adolescent depression, we found that connectivity between the left NAc and bilateral sgACC was negatively correlated with anhedonia severity^8^. Recent work in non-human primates has helped clarify the role of the ACC in anhedonia: chemically induced sgACC hyperactivity was shown to specifically blunt anticipatory, but not consummatory, arousal, while over-activation of the adjacent perigenual ACC had no effect^53^. Our current study is in excellent agreement with these findings, showing that shorter connectivity paths (E_Loc_) to the sgACC are associated with increased anticipatory, but not consummatory, anhedonia severity across a large cohort of clinically diverse adolescents.

Anticipatory anhedonia further correlated with the resting-state network properties of many key reward-related regions within RFT-derived networks. These included bilateral caudate E_Loc_ in the *Reward Anticipation* network, right OFC and dACC E_Loc_ in the *Reward Attainment* network, and bilateral caudate C_Str_/E_Loc_ as well as ventral striatum dACC E_Loc_ in the *Reward Prediction Error* network. The striatum and OFC are core components of the brain’s reward system, converting transient mesolimbic dopamine signals following primary rewards and reward-predicting cues into sustained representations of reward values and expectations^54^. The dACC is a functionally diverse region involved in reward valuation but also myriad aspects of negative affect, pain, cognitive control, and salience monitoring^55–57^. Single-neuron recordings in non-human primates indicate that these regions play complementary roles in reward processing, with striatum involved in learning to distinguish reward-predicting cues, the OFC encoding information about the type and magnitude of rewards, and the dACC predicting future rewards and detecting *Reward Prediction Errors*^54,58^. Interestingly, we found that subjects with higher anhedonia levels had increased network influence and communication efficiency in these reward-related regions, which is somewhat counterintuitive given the large body of animal studies and human fMRI research linking anhedonia to reduced reward activity^40,59,60^. However, it is important to note that resting-state functional connectivity and derived features like the graph theoretical metrics used in this study do not map neatly onto task activation patterns but rather reflect persistent interactions between brain regions^61^. If key reward processing areas have greater influence in adolescents with higher anhedonia levels, as the current study suggests, this could potentially exacerbate the effects of reduced reward activation reported in prior studies. Furthermore, our group has found reduced levels of gamma-aminobutyric acid (the main inhibitory neurotransmitter in the brain) in the ACC of depressed adolescents relative to healthy controls^9,62^, providing a potential mechanism for the observed hyperconnectivity.

It is also noteworthy that our correlation analyses revealed extensive associations between anticipatory anhedonia and graph theoretical metrics across different networks, while no associations were detected with consummatory anhedonia. Although this discrepancy may be due to our resting-state study design, which precluded active reward consumption, it suggests that network abnormalities in adolescents are disproportionately related to motivational impairments. Similarly, although we observed multiple associations with overall depression severity and especially the PVS construct of anhedonia, the negative valence system (NVS) construct of anxiety was not associated with any nodes or networks. The absence of significant correlations with overall anxiety severity may be due to our focus on networks derived from the RFT, which is specifically designed to interrogate PVS activation during different stages of reward processing^16^. However, we also observed relatively few regions that were correlated with total anhedonia or overall depression severity, suggesting that detailed symptom quantification improves power to detect associations with functional metrics that may be lost using generic clinical measures. Consistent with this, no group-level differences were found between adolescents with clinical symptoms vs. healthy controls in our main analysis, likely due to the heterogenous nature of the clinical cohort. Since categorical psychiatric diagnoses are often highly variable, we adopted an RDoC-style approach focusing on narrow, dimensional symptom measures instead.

Several caveats should be noted for this study. Foremost, although we recruited a relatively large cohort of 87 adolescents, sampling was more limited within major clinical categories of mood symptoms (*n* = 49), anxiety symptoms (*n* = 43), behavioral symptoms (*n* = 28), and especially healthy controls (*n* = 19). This study design was intended to capture the full range of clinical symptomatology by including subjects with significant comorbidity and subthreshold symptoms. As such, analyses focused primarily on associations with symptom severity in the full cohort; additional research is needed to determine how resting-state network properties differ between specific diagnostic groups and healthy adolescents. Second, although symptom severity is a more specific indicator of underlying PVS and NVS abnormalities than categorical diagnosis^6^, clinical symptoms are also heterogeneous to some extent. We were able to address this directly for anhedonia by separately analyzing anticipatory and consummatory TEPS subscales. To allow for comparably targeted analyses of depression and anxiety symptoms, our future work will employ more granular assessments, such as the behavioral inhibition and activation scales used by the Adolescent Brain Cognitive Development study to concisely assess the dimensions of goal-directed behavior, fun-seeking, reward responsiveness, and fearfulness^63^. We will also explore behavioral assessments, such as the Probabilistic Reward Task^64^, which can provide objective metrics of reward function and other clinically relevant capacities. Finally, although we used the best whole-brain parcellation currently available, there has been limited validation of the CAB-NP due to its recent release. However, all cortical boundaries were taken directly from the multimodal surface parcellation meticulously derived by the HCP^12^, which has been found to outperform other contemporary atlases and is widely considered a gold standard of human brain segmentation^65,66^. Subcortical parcels in the CAB-NP were then determined using a consensus partitioning approach based on data from over 300 HCP subjects divided into independent discovery and validation sets to ensure reproducibility and reliability^13^.

In conclusion, our study prioritized high-quality clinical and neuroimaging measures, recruiting a large cohort of psychotropic-medication-free adolescents to examine the full range of illness severity using sophisticated fMRI acquisition and analysis techniques. We found that PVS constructs of depression and anhedonia severity were associated with increased communication with key reward-related nodes in the medial PFC and striatum during rest. Conversely, no associations were observed between network communication metrics and clinical status or the NVS construct of anxiety. These results showcase the power of carefully constructed network models, data-driven analyses, and targeted clinical assessments to detect specific functional anomalies underlying emergent psychiatric symptoms. Identifying and characterizing these aberrant neurodevelopmental processes is crucial for understanding and ultimately stopping the course of mental illness.

## Supplementary information

Supplementary Materials

## Data Availability

Scripts used to automate calculation of graph theoretical metrics for this study are available at: https://github.com/elyb01/graph_theory_scripts_TP2021. All main and supplementary results data are freely available through the BALSA repository^38^ at: https://balsa.wustl.edu/study/show/x278x.
