# Supplementary material for: Data-driven parcellation and graph theory analyses to study adolescent mood and anxiety symptoms
Source: Transl Psychiatry. 2021 May 3;11:266. doi: 10.1038/s41398-021-01321-x (PMC8093238; doi:10.1038/s41398-021-01321-x)
Supplement: Supplementary file 1 — Supplementary Materials [file 41398_2021_1321_MOESM1_ESM.pdf]

Data-Driven Parcellation and Graph Theory Analyses to Study Adolescent Mood and Anxiety Symptoms

Benjamin A. Ely, PhD; Qi Liu, PhD; Samuel J. DeWitt, PhD; Lushna M. Mehra, BS;

Carmen M. Alonso, MD; Vilma Gabbay, MD

Supplementary Materials

Supplementary Methods

Construction of Reward Flanker Task (RFT) Networks

This study examined graph theoretical metrics within three reward-related networks derived from the RFT, described in detail in our previous studies.[1,2] The RFT was designed to distinguish neural activity during distinct phases of reward processing corresponding to the RFT networks used in the current study, i.e. reward anticipation, reward attainment, and reward prediction errors. Prior to fMRI, participants completed an RFT training session in a mock scanner to learn the rules of the task. In each RFT trial:

1) A cue was presented for 4-6s indicating how much money the participant could win in the current trial. Trial values consisted of high (50¢), low (10¢), no (0¢), or unknown (?) rewards. Outcomes for unknown cues were equally divided between high, low, and no rewards.
2) Flanker stimuli (e.g. “KKSKK”) were presented for 300ms.
3) Participants attempted to correctly identify the central letter while ignoring the flanking letters. Response windows were calibrated based on performance during training (max=1700ms).
4) Feedback (i.e. “Correct!”, “Incorrect!”, “Too Slow!”) was presented based on accuracy.

A total of 120 trials (30 per cue) were presented in pseudo-random order equally divided over four runs.

Subject-level RFT analysis was performed using Statistical Parametric Mapping (SPM)[3] v12 as part of a separate study in the same adolescent cohort (manuscript in preparation). Data preprocessing, multi-run ICA-FIX denoising, MSMAll alignment, and parcellation were identical to the procedures described in the main text **Methods**. GSR was not performed. Task data in CIFTI grayordinate space were temporarily converted to “pseudo-NIFTI” format for compatibility with SPM using Connectome Workbench v1.2.3.[4] General linear models were then constructed using 11 subject-level regressors: four for trial cues, three for correct feedback after certain (i.e. high/low/no) reward cues, three for correct feedback after unknown reward cues, and one for error (i.e. incorrect + too slow) feedback. Activation contrasts were constructed as:

1) *Reward Anticipation* = (high + low reward cues) – (no reward cues)
2) *Reward Attainment* = (high + low reward feedback) – (no reward feedback)
3) *Reward Prediction Error* = (feedback after unknown cues) – (feedback after certain cues)

The resulting subject-level contrast maps were converted back to CIFTI grayordinate format and combined at

the group level to create parcellated *t*-statistic maps using FSL PALM v111-alpha.[5] Finally, each group-level contrast map was thresholded at the 90th percentile to obtain a similar number of nodes (i.e. parcels) across contrasts. To avoid introducing artificial lateralization from arbitrary thresholding, each node was inspected and, if a corresponding contralateral node could be identified but was not already included in the network mask, the contralateral node was added. This yielded the final, symmetric network masks shown in main text **Figure 1**.

Supplementary Results

Group Difference and Symptom Correlations without Global Signal Regression (GSR)

Due to the large number of significant findings in our repeated analyses without GSR, results are presented in table format only. Note that **Table S2** (symptom correlations) is divided into five parts to keep related findings together while maintaining standard US page dimensions. Those interested in visualizing these results may download the complete dataset from our linked BALSA archive (https://balsa.wustl.edu/study/show/x278x).

Table S1: Graph Theory Group Contrast Results without GSR

**Network**	**Metric**	**Location**	**CAB-NP Label (HCP Label)** [a]	**Cohen *d*** [b]	p_{FWE}
Clinical > Control Adolescents					
Reward Anticipation	C_{Str}	Left Middle Frontal	Language_9 (55b)	0.910	0.030
	C_{Eig}	Left Middle Frontal	Language_9 (55b)	1.060	0.013
	E_{Loc}	Left Middle Frontal	Language_9 (55b)	0.826	0.047

[a] Labels per Cole-Anticevic Brain-wide Network Partition v1.0.5 (equivalent labels per HCP S1200 Release cortical parcellation).
[b] Adjusted for age and sex.

**Table S2 (part 1):** Graph Theory Symptom Correlation Results without GSR					
**Network**	**Metric**	**Location**	**CAB-NP Label (HCP Cortical Label)** [a]	**Pearson *r*** [b]	p_{FWE}
Depression Severity					
Reward Attainment	C_{Str}	Right Ventral Striatum	Cingulo-Opercular-3	0.399	0.014
		Right Inferior Pallidum	Orbito-Affective-17	0.372	0.031
	E_{Loc}	Right Ventral Striatum	Cingulo-Opercular-3	0.430	0.0053
Anticipatory Anhedonia Severity					
Whole Brain	C_{Str}	Left Superior Parietal	Dorsal_Attention_13 (MIP)	0.482	0.0071
		Right Lateral Parietal	Dorsal_Attention_10 (IP0)	0.460	0.017
		Left Lateral Temporal	Default_66 (TE1a)	0.455	0.022
		Left Lateral Occipital	Visual2_50 (V3CD)	0.432	0.041
		Right Lateral Occipital	Visual2_12 (LO2)	0.431	0.042
		Left Lateral Temporal	Default_64 (STSvp)	0.428	0.046
		Right Lateral Temporal	Default_28 (TE1a)	0.426	0.049
	E_{Loc}	Left Lateral Temporal	Default_66 (TE1a)	0.459	0.013
		Left Superior Parietal	Dorsal_Attention_13 (MIP)	0.455	0.014
		Left Lateral Occipital	Visual2_50 (V3CD)	0.444	0.018
		Right Lateral Temporal	Default_28 (TE1a)	0.437	0.024
		Right Lateral Parietal	Dorsal_Attention_10 (IP0)	0.436	0.026
		Right Lateral Occipital	Visual2_12 (LO2)	0.435	0.026
		Right Superior Parietal	Dorsal_Attention_2 (LIPd)	0.418	0.043
		Left Lateral Temporal	Default_64 (STSvp)	0.417	0.045
		Left Superior Parietal	Visual2_43 (VIP)	0.416	0.046
		Left sgACC	Default_74 (s32)	0.413	0.050
Reward Anticipation	C_{Str}	Right Lateral Occipital	Visual2_10 (V3B)	0.392	0.029
		Right Superior Parietal	Dorsal_Attention_2 (LIPd)	0.377	0.043
	E_{Loc}	Right Lateral Occipital	Visual2_10 (V3B)	0.386	0.023
		Right Superior Parietal	Dorsal_Attention_2 (LIPd)	0.378	0.028
		Right Lateral Occipital	Visual2_12 (LO2)	0.378	0.028
		Left Lateral Occipital	Visual2_37 (V3B)	0.375	0.031
		Left Middle Frontal Cortex	Language_9 (55b)	0.374	0.031
		Left Lateral Occipital	Visual2_39 (LO2)	0.371	0.034
		Left Superior Parietal	Dorsal_Attention_14 (LIPd)	0.364	0.040
Reward Attainment	C_{Str}	Right Lateral Parietal	Frontoparietal_21 (IP2)	0.409	0.026
		Left Lateral Temporal	Frontoparietal_44 (TE1p)	0.407	0.028
	E_{Loc}	Left sgACC	Default_74 (s32)	0.407	0.023
		Right Lateral Parietal	Frontoparietal_21 (IP2)	0.403	0.026
		Right sgACC	Default_6 (a24)	0.384	0.044
		Left dACC	Cingulo-Opercular_36 (33pr)	0.381	0.046
Reward Prediction Error	C_{Str}	Left Superior Parietal	Dorsal_Attention_13 (MIP)	0.495	<0.001
		Right Lateral Parietal	Dorsal_Attention_10 (IP0)	0.449	0.0039
		Right Lateral Occipital	Visual2_10 (V3B)	0.443	0.0050
		Left dlPFC	Language_15 (IFJa)	0.412	0.014
		Left Lateral Temporal	Default_64 (STSvp)	0.415	0.013
		Left Lateral Occipital	Visual2_50 (V3CD)	0.412	0.014
		Right Lateral Occipital	Visual2_12 (LO2)	0.401	0.020
		Right Superior Parietal	Dorsal_Attention_1 (MIP)	0.400	0.021
		Right Superior Parietal	Dorsal_Attention_2 (LIPd)	0.402	0.019
		Left Lateral Occipital	Visual2_37 (V3B)	0.394	0.025
		Right Lateral Occipital	Visual2_23 (V3CD)	0.371	0.046
		Left Lateral Parietal	Dorsal_Attention_22 (IP0)	0.371	0.047
		Left Superior Parietal	Dorsal_Attention_14 (LIPd)	0.368	0.050
*(continued in **part 2**)*					

Network	Metric	Location	CAB-NP Label (HCP Cortical Label) [a]	Pearson *r* [b]	p_{FWE}
**Table S2 (part 2):** Graph Theory Symptom Correlation Results without GSR					
Anticipatory Anhedonia Severity (con't)					
Reward Prediction Error (con't)	E_{Loc}	Left Superior Parietal	Dorsal_Attention_13 (MIP)	0.461	0.0014
		Left Superior PFC	Frontoparietal_41 (i6-8)	0.355	0.045
		Left dlPFC	Frontoparietal_32 (8C)	0.369	0.032
		Left dlPFC	Language_15 (IFJa)	0.411	0.010
		Left Lateral Temporal	Language_20 (STSdp)	0.352	0.048
		Left Lateral Temporal	Default_64 (STSvp)	0.419	0.0072
		Left Superior Parietal	Visual2_42 (LIPv)	0.396	0.016
		Left Superior Parietal	Dorsal_Attention_14 (LIPd)	0.395	0.016
		Left Lateral Parietal	Dorsal_Attention_22 (IP0)	0.397	0.016
		Left Lateral Occipital	Visual2_37 (V3B)	0.393	0.017
		Left Lateral Occipital	Visual2_50 (V3CD)	0.441	0.0035
		Left Lateral Occipital	Visual2_38 (LO1)	0.356	0.045
		Left Lateral Occipital	Visual2_39 (LO2)	0.400	0.015
		Left Lateral Occipital	Visual2_48 (V4t)	0.388	0.020
		Left Posterior Lateral Temporal	Visual2_27 (MST)	0.373	0.028
		Left Posterior Lateral Temporal	Visual2_44 (PH)	0.382	0.022
		Left Lateral Occipital	Visual2_31 (V4)	0.366	0.035
		Left Inferior Temporal	Visual2_53 (VVC)	0.419	0.0075
		Left Inferior Temporal	Visual2_47 (VMV3)	0.375	0.026
		Left Inferior Temporal	Visual2_32 (V8)	0.361	0.039
		Right Lateral Occipital	Visual2_12 (LO2)	0.417	0.0080
		Right Lateral Occipital	Visual2_10 (V3B)	0.423	0.0064
		Right Lateral Parietal	Dorsal_Attention_10 (IP0)	0.438	0.0038
		Right Superior Parietal	Dorsal_Attention_2 (LIPd)	0.407	0.012
		Right Superior Parietal	Dorsal_Attention_1 (MIP)	0.401	0.014
		Right Superior Parietal	Visual2_15 (LIPv)	0.378	0.025
		Right Lateral Occipital	Visual2_4 (V4)	0.373	0.028
		Right Lateral Occipital	Visual2_23 (V3CD)	0.403	0.013
		Right Lateral Occipital	Visual2_11 (LO1)	0.400	0.014
		Right Inferior Temporal	Visual2_13 (PIT)	0.375	0.026
		Right Inferior Temporal	Visual2_5 (V8)	0.374	0.026
		Right Inferior Temporal	Visual2_20 (VMV3)	0.358	0.043
		Right Posterior Lateral Temporal	Visual2_17 (PH)	0.382	0.022
		Right Posterior Lateral Temporal	Visual2 (MST)	0.360	0.040
		Right Lateral Temporal	Default_26 (STSvp)	0.383	0.022
		Right Lateral Occipital	Visual2_21 (V4t)	0.390	0.019
Consummatory Anhedonia Severity					
Whole Brain	C_{Str}	Right Cerebellum Lobule V	Posterior Multimodal-18	0.481	0.0084
		Left Inferior Parietal	Visual2_34 (V7)	0.443	0.029
	E_{Loc}	Right Cerebellum Lobule V	Posterior Multimodal-18	0.451	0.016
Reward Prediction Error	C_{Str}	Left Lateral Occipital	Visual2_37 (V3B)	0.394	0.026
		Left Superior Parietal	Dorsal_Attention_13 (MIP)	0.374	0.044
*(continued in **part 3**)*					

**Table S2 (part 3):** Graph Theory Symptom Correlation Results without GSR					
**Network**	**Metric**	**Location**	**CAB-NP Label (HCP Cortical Label)** [a]	**Pearson r** [b]	p_{FWE}
		Total Anhedonia Severity			
Whole Brain	C_{Str}	Left Superior Parietal	Dorsal_Attention_13 (MIP)	0.483	0.0088
		Left Lateral Temporal	Default_66 (TE1a)	0.449	0.027
		Left Lateral Temporal	Default_64 (STSvp)	0.438	0.038
		Left Superior Parietal	Visual2_43 (VIP)	0.456	0.022
		Left Superior Parietal	Visual2_35 (IPS1)	0.453	0.024
		Left Inferior Parietal	Visual2_34 (V7)	0.467	0.015
		Left Lateral Occipital	Visual2_37 (V3B)	0.458	0.021
		Left Lateral Occipital	Visual2_50 (V3CD)	0.456	0.022
		Right Lateral Parietal	Dorsal_Attention_10 (IP0)	0.470	0.014
		Right Lateral Occipital	Visual2_10 (V3B)	0.459	0.020
		Right Lateral Temporal	Default_28 (TE1a)	0.449	0.026
		Right Inferior Parietal	Visual2_8 (IPS1)	0.446	0.030
		Right Somatomotor Strip	Somatomotor_Leg (4-LL)	0.439	0.038
		Right Superior Occipital	Visual2_7 (V7)	0.437	0.039
		Right Lateral Occipital	Visual2_11 (LO1)	0.434	0.043
		Right Parahippocampus	Dorsal_Attention_6 (PHA3)	0.440	0.036
		Right Cerebellum Lobule V	Posterior Multimodal-18	0.473	0.013
	E_{Loc}	Left Lateral Occipital	Visual2_50 (V3CD)	0.468	0.0092
		Left Lateral Temporal	Default_66 (TE1a)	0.460	0.013
		Left Superior Parietal	Dorsal_Attention_13 (MIP)	0.456	0.015
		Left Inferior Parietal	Visual2_34 (V7)	0.455	0.015
		Right Lateral Temporal	Default_28 (TE1a)	0.456	0.015
		Right Lateral Temporal	Default_36 (STSva)	0.449	0.019
		Left vmPFC	Default_56 (10v)	0.450	0.019
		Right vmPFC	Default_18 (10v)	0.444	0.022
		Left Superior Parietal	Visual2_35 (IPS1)	0.448	0.020
		Left Superior Parietal	Visual2_43 (VIP)	0.451	0.018
		Left Lateral Occipital	Visual2_37 (V3B)	0.443	0.023
		Right Lateral Parietal	Dorsal_Attention_10 (IP0)	0.447	0.020
		Right Parahippocampus	Dorsal_Attention_6 (PHA3)	0.450	0.019
		Right Parahippocampus	Default_32 (PHA2)	0.419	0.047
		Right Inferior Parietal	Visual2_8 (IPS1)	0.438	0.026
		Right Lateral Occipital	Visual2_10 (V3B)	0.439	0.026
		Right Superior Occipital	Visual2_7 (V7)	0.431	0.033
		Right Lateral Occipital	Visual2_11 (LO1)	0.438	0.027
		Right Lateral Occipital	Visual2_12 (LO2)	0.427	0.038
		Left Lateral Occipital	Visual2_39 (LO2)	0.434	0.030
		Right Lateral Occipital	Visual2_23 (V3CD)	0.422	0.044
		Left Lateral Parietal	Default_68 (PGi)	0.431	0.033
		Left Lateral Temporal	Default_64 (STSvp)	0.436	0.029
		Left Temporal Pole	Language_17 (STGa)	0.418	0.047
		Left Lateral Temporal	Default_75 (STSva)	0.421	0.044
		Right Somatomotor Strip	Somatomotor_Leg (4-LL)	0.436	0.029
		Left Superior Parietal	Visual2_42 (LIPv)	0.417	0.049
		Right Superior Parietal	Dorsal_Attention_1 (MIP)	0.421	0.044
		Left sgACC	Default_74 (s32)	0.420	0.045
		Right Cerebellum Lobule V	Posterior Multimodal-18	0.466	0.010
		Right Hippocampus	Visual2-25	0.419	0.046
Reward Anticipation	C_{Str}	Right Lateral Occipital	Visual2_10 (V3B)	0.405	0.021
		Left Lateral Occipital	Visual2_37 (V3B)	0.385	0.035
	E_{Loc}	Left Lateral Occipital	Visual2_39 (LO2)	0.382	0.024
		Right Lateral Occipital	Visual2_10 (V3B)	0.379	0.026
		Left Lateral Occipital	Visual2_37 (V3B)	0.378	0.027
		Left Middle Frontal	Language_9 (55b)	0.357	0.047
		Right Lateral Occipital	Visual2_12 (LO2)	0.358	0.046
		*(continued in **part 4**)*			

**Table S2 (part 4):** Graph Theory Symptom Correlation Results without GSR					
**Network**	**Metric**	**Location**	**CAB-NP Label (HCP Cortical Label) [a]**	**Pearson *r* [b]**	p_{FWE}
		Total Anhedonia Severity (con't)			
Reward Attainment	C_{Str}	Right Inferior Parietal	Frontoparietal_21 (IP2)	1.335	0.046
	E_{Loc}	Left sgACC	Default_74 (s32)	0.398	0.028
		Left sgACC	Default_46 (10r)	0.389	0.036
		Left Lateral Occipital	Visual2_33 (V3A)	0.395	0.031
		Right Lateral Occipital	Visual2_6 (V3A)	0.379	0.047
Reward Prediction Error	C_{Str}	Left Superior Parietal	Dorsal_Attention_13 (MIP)	0.494	<0.001
		Right Lateral Occipital	Visual2_10 (V3B)	0.460	0.0037
		Right Lateral Parietal	Dorsal_Attention_10 (IP0)	0.447	0.0056
		Left Lateral Occipital	Visual2_37 (V3B)	0.445	0.0058
		Left Lateral Temporal	Default_64 (STSvp)	0.435	0.0074
		Right Superior Parietal	Dorsal_Attention_1 (MIP)	0.432	0.0078
		Left Lateral Occipital	Visual2_50 (V3CD)	0.413	0.014
		Left Lateral Occipital	Visual2_39 (LO2)	0.393	0.025
		Right Lateral Occipital	Visual2_11 (LO1)	0.394	0.024
		Left dlPFC	Language_15 (IFJa)	0.389	0.028
		Left dlPFC	Frontoparietal_32 (8C)	0.381	0.036
		Right Lateral Occipital	Visual2_12 (LO2)	0.384	0.032
		Right Posterior Lateral Temporal	Visual2 (MST)	0.378	0.039
		Right Posterior Lateral Temporal	Visual2_17 (PH)	0.372	0.046
		Right Lateral Temporal	Default_26 (STSvp)	0.382	0.035
		Left Lateral Occipital	Visual2_38 (LO1)	0.371	0.047
		Left Superior Parietal	Visual2_42 (LIPv)	0.369	0.049
		Left Lateral Parietal	Dorsal_Attention_22 (IP0)	0.371	0.047
		*(continued in **part 5**)*			

**Table S2 (part 5):** Graph Theory Symptom Correlation Results without GSR					
**Network**	**Metric**	**Location**	**CAB-NP Label (HCP Cortical Label)** [a]	**Pearson *r*** [b]	p_{FWE}
Total Anhedonia Severity (con't)					
Reward Prediction Error (con't)	E_{Loc}	Left Superior Parietal	Dorsal_Attention_13 (MIP)	0.448	0.0036
		Left Lateral Occipital	Visual2_50 (V3CD)	0.434	0.0050
		Left Lateral Temporal	Default_64 (STSvp)	0.432	0.0053
		Right Lateral Occipital	Visual2_10 (V3B)	0.430	0.0055
		Right Lateral Parietal	Dorsal_Attention_10 (IP0)	0.423	0.0079
		Right Superior Parietal	Dorsal_Attention_1 (MIP)	0.419	0.0090
		Left Lateral Occipital	Visual2_37 (V3B)	0.417	0.0092
		Right Lateral Occipital	Visual2_11 (LO1)	0.413	0.011
		Right Lateral Temporal	Default_26 (STSvp)	0.402	0.014
		Left Lateral Occipital	Visual2_39 (LO2)	0.406	0.013
		Right Posterior Lateral Temporal	Visual2 (MST)	0.399	0.015
		Right Lateral Occipital	Visual2_23 (V3CD)	0.397	0.016
		Left Superior Parietal	Visual2_42 (LIPv)	0.394	0.017
		Right Superior Parietal	Visual2_15 (LIPv)	0.386	0.020
		Left Inferior Temporal	Visual2_53 (VVC)	0.395	0.017
		Left Inferior Temporal	Visual2_47 (VMV3)	0.378	0.023
		Left Lateral Occipital	Visual2_31 (V4)	0.375	0.025
		Right Lateral Occipital	Visual2_4 (V4)	0.372	0.027
		Left dlPFC	Language_15 (IFJa)	0.391	0.018
		Right Posterior Lateral Temporal	Visual2_17 (PH)	0.381	0.021
		Left dlPFC	Frontoparietal_32 (8C)	0.383	0.021
		Left Lateral Occipital	Visual2_38 (LO1)	0.387	0.020
		Left Lateral Parietal	Dorsal_Attention_22 (IP0)	0.384	0.021
		Left Lateral Occipital	Visual2_48 (V4t)	0.389	0.019
		Left Posterior Lateral Temporal	Visual2_27 (MST)	0.386	0.020
		Right Lateral Occipital	Visual2_12 (LO2)	0.388	0.019
		Right Lateral Occipital	Visual2_21 (V4t)	0.382	0.021
		Left Lateral Temporal	Language_20 (STSdp)	0.379	0.023
		Left Superior Parietal	Dorsal_Attention_14 (LIPd)	0.369	0.030
		Left Posterior Lateral Temporal	Visual2_44 (PH)	0.369	0.029
		Left Inferior Temporal	Visual2_32 (V8)	0.369	0.030
		Right Inferior Temporal	Visual2_20 (VMV3)	0.368	0.031
		Right Inferior Temporal	Visual2_5 (V8)	0.359	0.037
		Right Inferior Temporal	Visual2_13 (PIT)	0.354	0.043
		Right Lateral Temporal	Language_6 (STSdp)	0.349	0.048
		Right Superior Parietal	Dorsal_Attention_2 (LIPd)	0.360	0.036

[a] Labels per Cole-Anticevic Brain-wide Network Partition v1.0.5 (equivalent labels per HCP S1200 Release cortical parcellation).
[b] Adjusted for age and sex. Anhedonia correlations reported with negative TEPS for consistency with other scales (see **Methods**).

Supplementary References

1 Bradley, K. A. L., Case, J. A. C., Freed, R. D., Stern, E. R. & Gabbay, V. Neural correlates of RDoC reward constructs in adolescents with diverse psychiatric symptoms: A Reward Flanker Task pilot study. *J Affect Disord* **216**, 36-45, doi:10.1016/j.jad.2016.11.042 (2017).

2 Bradley, K. A. *et al.* Relationships between neural activation during a reward task and peripheral cytokine levels in youth with diverse psychiatric symptoms. *Brain Behav Immun* **80**, 374-383, doi:10.1016/j.bbi.2019.04.014 (2019).

3 Friston, K. J. *et al.* Statistical parametric maps in functional imaging: a general linear approach. *Hum Brain Mapp* **2**, 189-210, doi:10.1002/hbm.460020402 (1994).

4 Marcus, D. S. *et al.* Human Connectome Project informatics: quality control, database services, and data visualization. *NeuroImage* **80**, 202-219, doi:10.1016/j.neuroimage.2013.05.077 (2013).

5 Winkler, A. M., Ridgway, G. R., Webster, M. A., Smith, S. M. & Nichols, T. E. Permutation inference for the general linear model. *NeuroImage* **92**, 381-397, doi:10.1016/j.neuroimage.2014.01.060 (2014).
